# Suggestions Regarding the Obligations of the Dentist to Himself

**Published:** 1903-12

**Authors:** F. Milton Smith


### SUGGESTIONS REGARDING THE OBLIGATIONS OF THE DENTIST TO HIMSELF.[1]

BY F. MILTON SMITH, D.D.S.

Some time since it was reported that a well-known dentist of this country stated publicly that his first thought on examining a mouth is, "How much is there in it for me?" The writer does not believe this expression represents fairly the thought and purpose of a majority of practitioners. On the contrary, he is persuaded that the average dentist is desirous of doing the best he possibly can for his patient, and that with the minimum of expense, always remembering that "the laborer is worthy of his hire." That the average dentist would deliberately fill a tooth with gold when he believed gutta-percha or amalgam would do as well, simply because he could get a larger fee, the writer does not believe. Assuming, then, that the dentist has a higher ambition than the securing of gold for his coffers, let us consider some of the obligations under which he rests as regards his duty to himself; for if he shall be true to himself, and fair in his treatment of others, he should incidentally secure sufficient of this world's goods to supply the daily needs of himself and those dependent upon him, with some provision for a rainy day.

Since the close confinement of the busy dentist is not conducive to the best physical condition, it is his duty to see to it that the ill effects likely to result therefrom are counteracted so far as possible by every available means. If Drs. R. W. Varney and Marshal H. Webb had conserved their energies and husbanded their resources, they would probably both have lived years longer to have blessed humanity. They are, however, sad illustrations of a class of worthy men, too many of whom are found to-day, who are taxing themselves far beyond the point indicated by prudence and sound judgment. A very dear friend said to the writer some months since, "I have for ten years averaged ten hours a day for ten months in the year, besides setting up many nights till one and two o'clock writing." Another friend, whose appointment-book the writer looked over, is working almost every minute in the day from 8.30 to 5.30 or six o'clock, with a very few minutes for lunch.

---

[1] Read before The New York Institute of Stomatology, June 2, 1903.

The latter told the writer that he was considering spending his vacation time in undergoing, and maybe recovering from, an operation for appendicitis, with which trouble a prominent surgeon advises him he is suffering. The former gentleman has already undergone two or three very serious surgical operations, which in the opinion of the writer might have been avoided had he applied himself less closely to business. The dentist is likely to work more years if he works not more than seven hours in the day in the office, and takes a vacation of at least four weeks each year. Some will say it is necessary to work more than seven hours in order to make both ends meet. The answer would be, if one's time is fully taken up for more than seven hours, at certain fees, it would be wiser, judiciously to increase the fees rather than to work more hours. When a dentist has practised sufficiently long to have accumulated a practice that keeps him occupied steadily for seven hours each day, his time, if he has been faithful, should return him more than barely sufficient for his daily needs. In the opinion of the writer, one reason why the conscientious dentist is often unable to get along without working nine or ten hours each day is, the people have been taught that a bill should represent a certain number of cavities filled, with little or no consideration for that which should be compensated for beyond the other,—namely, professional skill and service. I will guarantee that there are good men in this room who would be actually ashamed to have the truth known as to the fees charged by them for such a service as is involved in properly treating and filling an abscessed tooth of three or four roots, to say nothing of cleansing and polishing, which, if properly done, is one of the most valuable services we can render.

Your essayist has many times been surprised at the amount of hedging that is seemingly done in the matter of fees. In many cases a charge of next to nothing is made for what the dentist knows is the most valuable service he has rendered, with a compensating charge of five dollars each for filling six small cavities with gold; the gold fillings having taken less time to make, and infinitely less skill, than the service for which no charge is made. It would seem more honorable and self-respecting to say to the patient, if items are given at all, "The treatment of the abscessed tooth costs you thirty dollars, the gold fillings I will make you a present of." While the writer does not always succeed in convincing a new patient that he is not a highway robber, he does try to convince him that his bill is for honest professional service, and not for a few pieces of gold-foil. He believes this is one of the principal obligations a dentist is under to himself and to the profession.

The writer has no warmer friends nor more appreciative patients in his practice than those who have submitted to be treated in a strictly professional manner in this respect. They are saved the annoyance of remembering how much the last gold filling cost, and comparing it with the size of the one put in to-day. They can, indeed, some of them, make out their own bill when they leave the chair. The writer does not believe he could do a beginner a better service, in its bearing upon his whole life from a business standpoint, than to persuade him to make his charges upon a time basis, even if he finds it necessary to make his fee as low as one dollar per hour to begin with. The system of charging by the cavity seems to the writer most pernicious in its effects. The young man who does it will need to be of unusually good moral fibre if he does not at times place three or four small fillings where a single substantial one should be made.

Were it not for the fact that one of our best men said to the writer some time since, "I charge by the hour, but do not let my patient know it," the matter would have received less attention. The writer has sometimes wondered whether older men than beginners do not put amalgam in some large cavity because their fee, as announced by the cavity, is not sufficient to compensate them for time spent in making a large gold filling.

Returning, however, to the duty of the dentist to care for his health, we would suggest the wisdom of looking carefully after the eyes, which to him are so important. The writer for years labored under an unnecessary burden in working without glasses while suffering with a pronounced astigmatism, the existence of which he was totally ignorant of until he found that a full day's work exhausted him far beyond that which it should at thirty years of age. As he now remembers it, a word of caution in an article by Dr. S. G. Perry first aroused his suspicions that his eyes were at fault. The writer has found much relief, as to strain upon his eyes, in using an opaque curtain to shut out all light coming through the window from below a line with the head-rest of the chair.

The dentist should study to make himself as comfortable at the chair while operating as possible. Operators who have not learned to operate while looking in the glass at the reflected tooth cannot imagine the great relief that is experienced in so doing. The writer has derived much satisfaction and comfort from using an instrument combining a mirror and a point to hold the first pieces of gold in a cavity. By its aid he is able to fill all ordinary approximal cavities in the incisor teeth from the palatal side while standing nearly erect over the patient.

It is the invention of Dr. Hodson, of Thirty-ninth Street, this city, and was formerly made by the S. S. White Company. The writer was recently informed that the company had ceased the manufacture of them because the dentists did not appreciate their usefulness. The writer is fully persuaded, from his limited experience in the use of a stool for operating, that it is the duty of every young man to accustom himself to its use. He is confident that, if it had been called earlier to his attention, it would have been better for him.

The employment of a female assistant cannot be too strongly recommended, and she should be taught to do everything that it is possible to delegate to her. The dentist who administers a general anæsthetic is likely to subject himself and his patient to great embarrassment if he does not have a female assistant present during such administration. He may even find it extremely difficult to controvert the suggestion that may have come to the mind of his patient while under the influence of the anæsthetic, and he thus be held under suspicion of that of which he is entirely innocent. Since the condition of the mind has much to do with the physical condition, the dentist should protect himself from those petty annoyances which will occur unless guarded against. It would be better to give one-half hour in the day for examinations, consultations, etc., without charge than to permit these to annoy one at irregular intervals through the day; to say nothing of the injustice of keeping the patient waiting who is paying for our time at the chair. The assistant, if she has tact, will do much to relieve us in this direction.

"Lest we forget," may we call attention to the almost universal habit of dentists of neglecting their own teeth, also to the indifferent class of dental service usually rendered by dentists to their *confrères.* The writer has heard his own mouth spoken of as one representing the best class of dental work, as a whole, ever seen (by the dentist making the remark) in the mouth of a dentist.

This is attributable, first, to good luck in having, as a boy, fallen into good hands; and second, to the fact that he has made appointments with good dentists, kept his appointments, submitted to being a patient, and paid for his work. The average dentist runs in on his friend when he happens to have a few moments of leisure, and has some slipshod plastic work done, with the promise that later on he will make appointments and have his mouth put in order, which he never does, hence exposed pulps, alveolar abscesses, pyorrhœa, and foul breath abound in the mouth of the dentist, "who, having preached to others, becomes himself a castaway."

The dentist should not combine home and business under the same roof. There are two principal reasons for this: First, his patients will expect him to be always at their service to operate for them at unreasonable times in the morning and evening, to say nothing of Sundays and holidays. Second, the dentist will be much more liable to suffer from lack of out-door exercise. If possible he should live so far as to necessitate a walk of a mile or two morning and evening, and this walk should be a brisk one. Better than this would it be if he were so situated that he could ride a bicycle at least ten miles a day.

Your essayist, for more than thirty years, averaged two weeks out of every year ill in bed. During the years of 1899 and 1900 he rode five thousand miles on a wheel, for the most part from home to office and return, with the result, no day in bed in two years.

Whether it be walking, bicycling, horseback riding, or what not, of one thing make sure,—the dentist who does not get some out-of-door exercise will pay for it in days cut off from his life. Let it be remembered that one of the largest debts a dentist owes himself is that he shall find some wholesome diversion that will enable him at times to forget he is a dentist.

If a dentist will make anything of a financial success of his life he must learn self-denial, must be methodical, must waste no time, and must keep his word and credit good. He must be business-like, and send his bills regularly and promptly, and he should know where his money goes. He must keep abreast of the times by reading the journals carefully, and he must prepare himself to do not only the ordinary, every-day work, but the work that is considered unusual.

The dentist who finds it necessary to refer every third patient to some other man is not doing for himself all he could. Furthermore, let him remember that it is his business to make a favorable impression upon those with whom he comes in contact, always keeping in mind the thought of one who wrote, "This above all: to thine own self be true, and it must follow, as the night the day, thou canst not then be false to any man."

